# Mixed Neuroendocrine Non-Neuroendocrine Tumor (MINEN) of the Liver: Report of Two Cases and Review of the Literature

**DOI:** 10.5146/tjpath.2024.13492

**Published:** 2025-01-31

**Authors:** Basharat Mubeen, Malini Eapen, S. Sudhindran, Nikhil Krishna Haridas

**Affiliations:** Department of Pathology, Amrita Institute of Medical Sciences, Kerela, India; Department of Surgical Gastroenterology, Amrita Institute of Medical Sciences, Kerela, India; Department of Clinical Hematology, Stem Cell Transplantation and Medical Oncology, Amrita Institute of Medical Sciences, Kerela, India

**Keywords:** Liver, MINEN, Neuroendocrine, Non-neuroendocrine

## Abstract

***Objective: *** To highlight two cases mixed neuroendocrine non-neuroendocrine tumors (MINEN) of the liver and to review the literature till date.

To present two cases of MINEN of the liver diagnosed in our centre with clinical & diagnostic workup, the treatment modalities, and follow up. Extensive review of the literature and compilation of the presentation and treatment modalities used in those cases.

***Case Reports: *** Thirty-three cases of MINEN of the liver have been reported till date including ours. Our cases presented as incidental masses in liver during workup for other symptoms. AFP levels were normal in both cases but PIVKA (Protein induced by vitamin K absence) levels were increased. Resection was done in one of the cases while the other patient had to undergo transplantation. A diagnosis of MINEN was made on H&E, and confirmed on IHC. One patient was unfit for systemic chemotherapy whereas the other patient received cisplastin and etoposide based chemotherapy. Both patients developed metastasis on follow up but are still alive after 12-15 months.

***Conclusion: *** MINEN is an uncommon tumor of the liver with a poor prognosis as shown by the few studies available. Recurrence and distant metastases are often described even after complete resection and the course is fatal. The role of adjuvant chemotherapy following surgical resection is not fully elucidated. Mean survival in the cases reported ranged from 1 month to 33 months. However, no significant differences were seen in the clinicopathologic profile of the cases described so far. Further multiinstitutional studies and follow up will help to further characterize this subtype for appropriate treatment.

## INTRODUCTION

Mixed hepatocellular carcinoma-neuroendocrine carcinomas (HCC-NECs) are rare, and they usually carry a poor prognosis (1). Mixed HCC-NECs account for 0.4% of primary hepatic tumors (2). As per the latest guidelines, the mixed neuroendocrine non neuroendocrine (MINEN) component has a a neuroendocrine carcinoma (NEC) component and non-NEC component (hepatocellular or cholangiocarcinoma), each of which is histologically and immunohistochemically recognizable as a discrete component and accounts for >30% of the neoplasm (3). In this paper we report two patients who presented with incidental masses in the liver during workup of cirrhosis. AFP levels were normal in both cases but PIVKA (Protein induced by vitamin K absence) levels were significantly increased. Resection with curative intent was done in one of the cases while the other patient underwent liver transplantation. A diagnosis of MINEN was suspected on light microscopy, and confirmed on immunohistochemistry (IHC). We have also reviewed the literature of all the cases reported till date including the various treatment modalities used. However, overall survival and prognosis of these cases remains grim.

### Case Report 1

**Figure 1:**
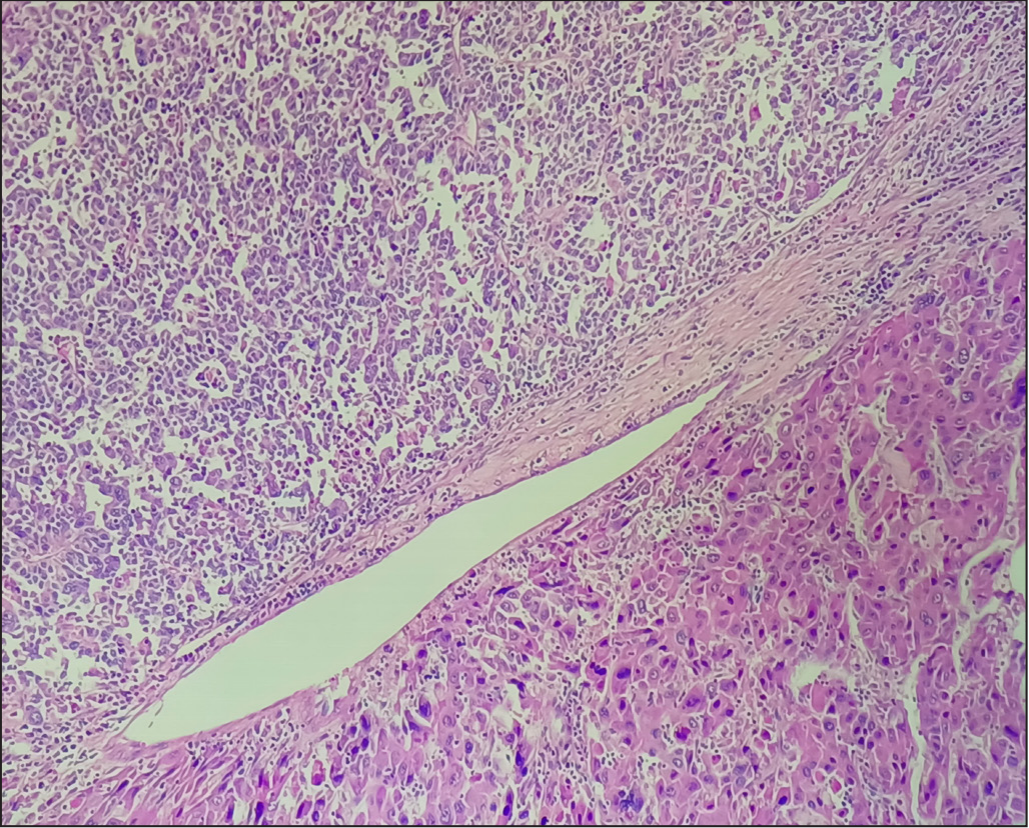
Microscopic sections showing a sharp demarcation between pleomorphic HCC component and small cell neuroendocrine component separated by a thin fibrous septae focally (H&E, 20x).

**Figure 2:**
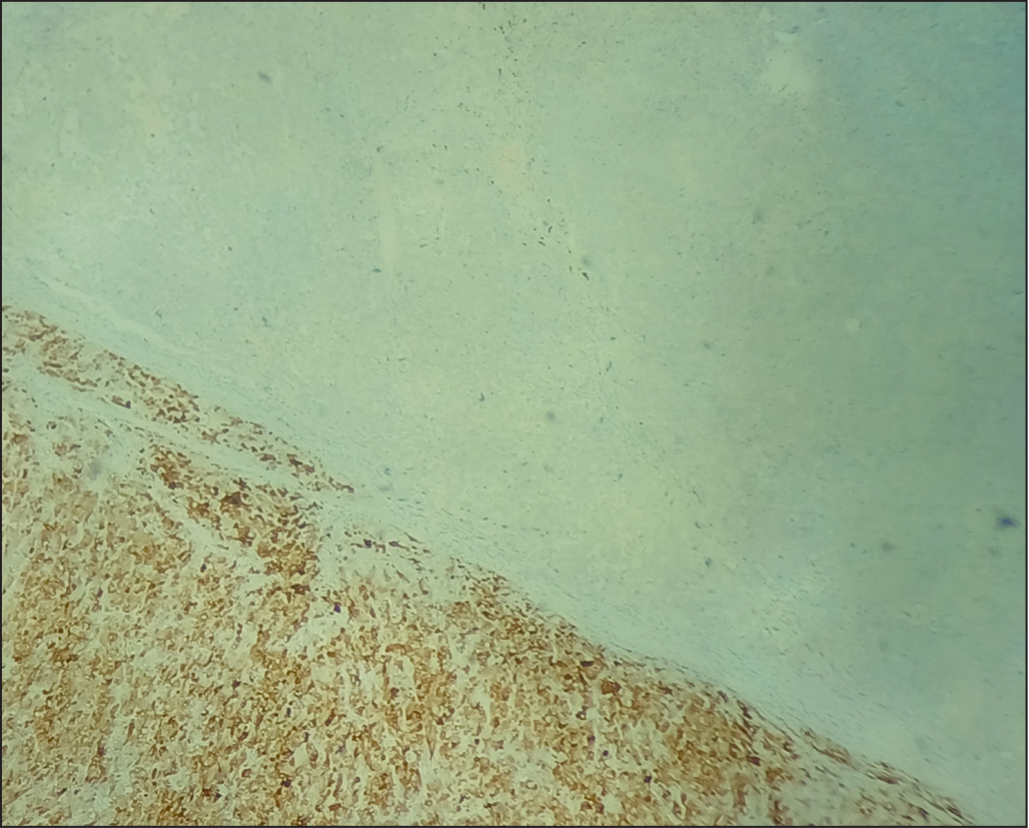
Arginase highlighting the HCC component and was negative in neuroendocrine component (Arginase, 20x).

**Figure 3:**
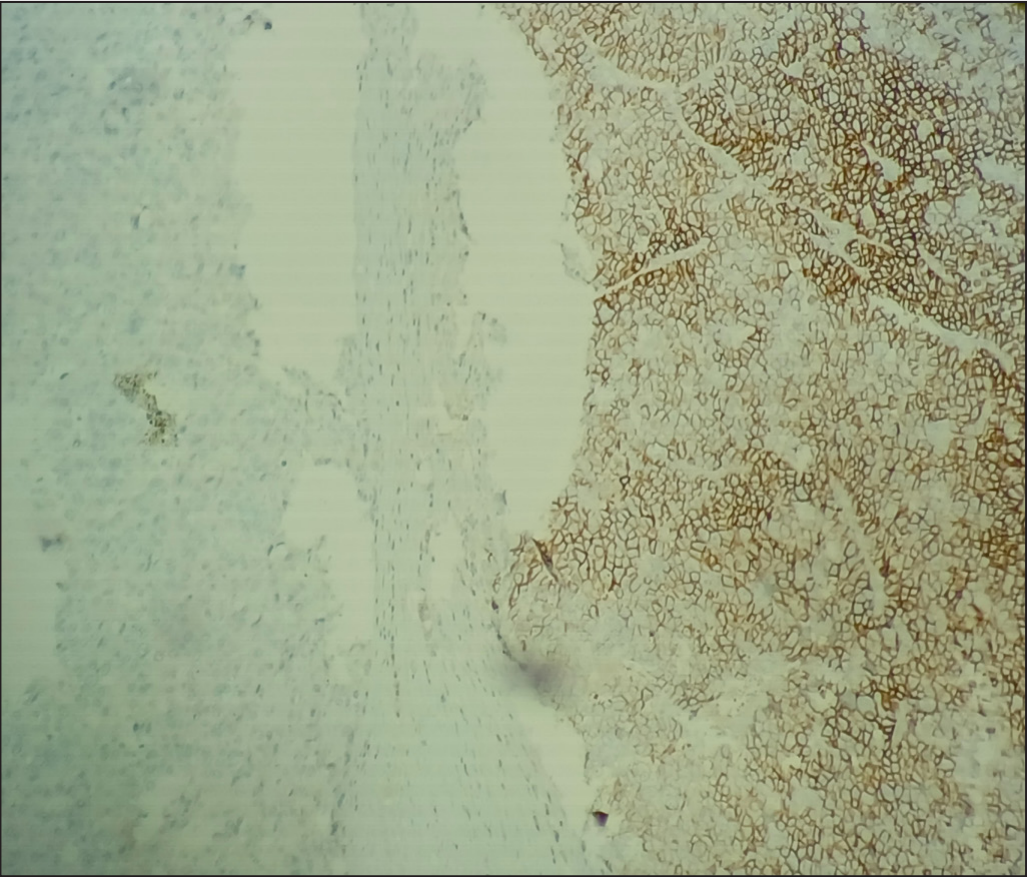
Synaptophysin showing membranous positivity in the neuroendocrine component and was negative in the HCC component, with a sharp demarcation between two components (Synaptophysin, 20x).

**Figure 4:**
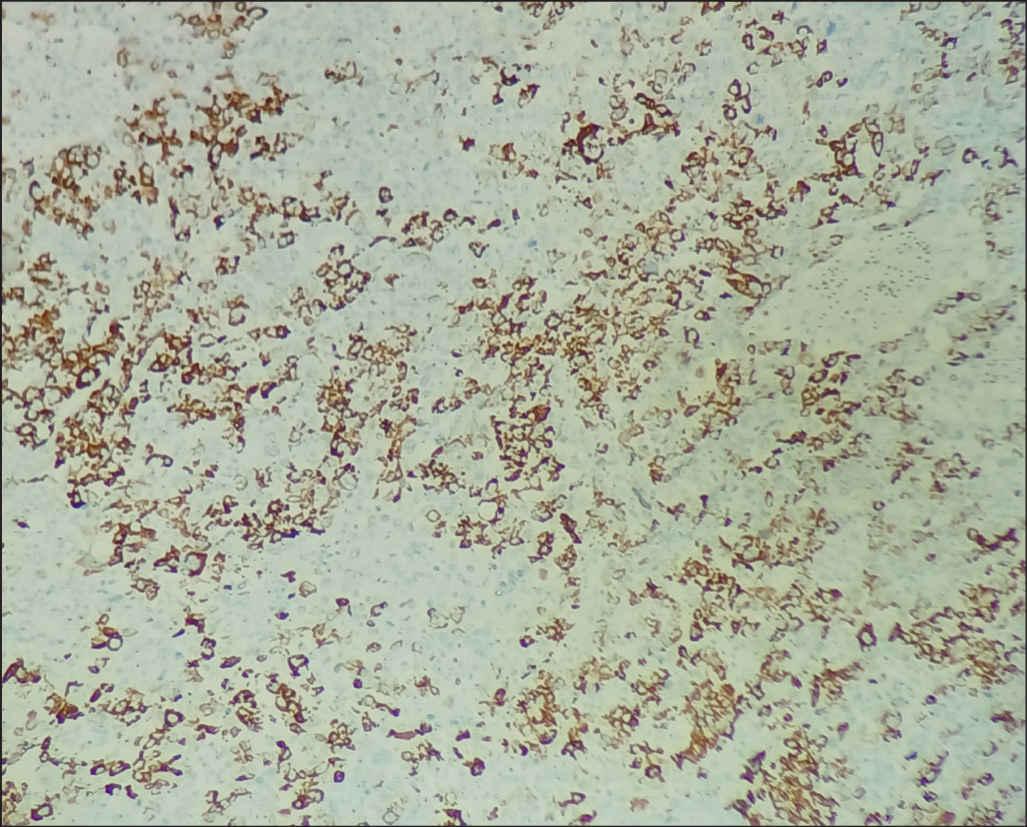
Synaptophysin highlighting the transitional zone, where two components were intimately admixed (Synaptophysin, 20x).

A 73-year-old male with multiple comorbidities like systemic hypertension, diabetes mellitus, and distal lymphedema was evaluated for diarrhea. Incidentally a liver lesion was detected on USG and MRI abdomen in segment 7 with features suggesting HCC. No definite vascular invasion was noted. Patient was referred to our centre for further evaluation. PIVKA levels were 1579.63 mAU/ml and AFP levels 21.20 ng/ml. His upper gastrointestinal endoscopy was normal. No features of portal hypertension were noted on oesophagogastroduodenoscopy. He underwent diagnostic laparoscopy and non-anatomical liver resection. Resection of segments 7 and 8 of the liver was done with curative intent. In our department we received segment 7 & 8 hepatectomy specimen measuring 10x12.8x4 cm. On serial slicing, an unifocal lesion that was well circumscribed, encapsulated, and lobulated was seen with a grey white to tan brown cut surface that was soft in consistency. The lesion measured 4x5x5 cm. No areas of necrosis and fibrosis were identified. No satellite nodules or vascular plugs were noted. The adjacent liver appeared unremarkable. Histology sections from the liver showed an irregularly circumscribed neoplasm with a thick and thin fibrous capsule at the interface and was arranged in nodules separated by hyalinized fibrocollagenous septae. Within the nodules, cells were arranged in acinar, trabecular, diffuse, solid, peritheliomatous and focal rosetting pattern with areas showing dilated sinusoids. Individual cells varied from medium to large with eosinophilic cytoplasm and vesicular nuclei with nucleoli. Pleomorphic, multinucleate, rhabdoid and bizarre cells were seen. Focal areas showed cells with small hyperchromatic nuclei and rosettoid pattern. This area showed increased mitosis as compared to surrounding pleomorphic HCC. These two patterns were intermixed at places and had a sharp demarcation at some foci (Figure 1). Various differential possibilities like mixed HCC and cholangiocarcinoma, HCC with stem cell features and MINEN (HCC with neuroendocrine carcinoma) were considered. No vascular invasion or perineural invasion was noted. The margins were free of tumor. Clearance from the closest inked margin was 1 mm. The adjacent liver showed features of incomplete septal cirrhosis. On IHC, Arginase (Figure 2) and Hep par was positive in some of the neoplastic nodules. Glypican was focally positive. CD 10 showed cytoplasmic and membranous positivity in all neoplastic cells. CD 34 showed capillarisation of sinusoids. No vessels encircling tumor cells (VETC) pattern was seen on CD34 immunostain. CK 19, which has prognostic and diagnostic significance, was positive in many cells. Monotonous small cell areas described in histology with acinar/rossettoid pattern showed synaptophysin (Figure 3, Figure 4) and CD 56 positivity. Ki-67 proliferative index was 40-45% in the HCC component and 80% in the small cell component. CK 7, Chromogranin, AFP, CEA and CD 117 were negative. Finally a diagnosis of MINEN (Grade III HCC and neuroendocrine carcinoma) was rendered. The post operative period was uneventful. Post operative CT showed a tiny nodule in the lung (probably metastasis). In view of the small cell carcinoma component, a decision of systemic chemotherapy was made. However, the patient was unfit for systemic therapy on immediate follow up. The patient was counseled regarding the prognosis and disease outcome and a decision was made to reassess him after 3 months. After 5 months, PET CT was done and revealed an increase in the size of the lung nodule. Bilateral FDG avid adrenal lesions were seen, suggestive of metastasis. Multiple paratracheal nodes were also noted, which turned out to be reactive on endobronchial ultrasound-guided fine needle aspiration biopsy. In view of adrenal metastasis, patient was scheduled for external beam radiotherapy scheduled as 900cGy per fraction with 5 fractions a week. At 14 months, the patient is alive.

### Case report 2

A 44-year-old female known to have Type 2 diabetes, premorbid obesity, and bronchial asthma presented with pedal edema and ascites. On evaluation, she was found to have cirrhosis of the liver with a mass in segment 6 and 7. AFP levels were 7.02 ng/ml and PIVKA levels were 236 mAU/ml. Her model for end stage liver disease (MELD) score was 22. In view of the high MELD score and liver mass showing arterial enhancement and delayed venous outflow suggestive of HCC, a decision of living donor liver transplantation was made. We received a 22 x 10.5 x 6 cm liver explant. The capsule was intact. On serial slicing, multifocal tumors were noticed. Lesion I in the right lobe measured 7 x 4 x 3 cm. A nearby satellite nodule measured 0.5 x 0.8 x 0.5 cm. It was 0.5 cm away from lesion I. Another satellite nodule was 1.5 cm away from this lesion and measured 0.3 x 0.4 x 0.5 cm. Lesion II was in the subcapsular region of the right lobe and measured 1 x 0.5 x 1 cm. Lesion III measured 0.7 x 0.7 x 1.5 cm. Lesion IV measured 1.2 x 0.7 x 1.3 cm. Lesion V measured 0.8 x 0.8 x 0.5 cm. Lesion VI measured 0.8 x 0.8 x 0.6 cm. Histology showed high-grade HCC areas admixed with small to medium sized cells arranged in gyriform, trabeculae and rosettoid patterns with scanty cytoplasm and uniform round to ovoid vesicular nucleus with fine chromatin and high N/C ratio, and showing mitoses and apoptosis. A diagnostic possibility of mixed HCC and neuroendocrine carcinoma was made and IHC confirmed positivity for arginase and HepPar1 in HCC-like areas and synaptophysin, chromogranin and INSM1 in the neuroendocrine component. The Ki-67 index was 67-90% in the neuroendocrine areas. CD117 was negative, ruling out HCC with stem cell features. Finally a possibility of multifocal HCC with neuroendocrine carcinoma (MiNEN)- combined type was made. The patient was scheduled for adjuvant cisplatin & etoposide (EPO) regimen of 4-6 cycles. During follow-up, the patient developed an extradural mass lesion at D6-D8, likely a metastasis. At 1 year, the patient is alive.

## DISCUSSION

All types of primary neuroendocrine tumors (NET) of the liver are very rare and account for 0.4% of the liver tumors. Most of them are actually metastatic rather than primary (3). So before accepting NET as a primary, the possibility of metastasis needs to be ruled out. The possibility of neuroendocrine tumor to be part of a mixed tumor (MINEN) is slightly more than primary neuroendocrine tumors of liver and accounts for roughly 0.5% (2,4). Jahan et al. (4) did a thorough review of the literature from 1970 to 2020, and they found around 28 cases of mixed neuroendocrine and non-neuroendocrine tumors of liver. Afterwards Lan et al. (5) published one case in 2021 and also Tanaka et al. (6) in 2022. The latest case published was in 2023 by Shin (7). Hence the total becomes 33 including both our cases, highlighting the rarity of this tumor. All the cases cited in the literature are tabulated in Table 1. Twenty-one out of 33 (63.3%) of cases were reported from Asia, coinciding with the geographic distribution of HCC (4). The male to female ratio was 10:1. Only 3 female patients were reported (8,9) in the literature including one of our cases. A literature review by Lan et al. (5) found 12 cases out of 28 (42.8%) having cirrhosis. Chronic HBV/HCV infection turns out to be the most significant risk factor. Out of 33 patients cited in the literature, HBV/HCV infection was present in 22 cases (66.66%). Our patients were negative for HBV/HCV.

**Table 1:** Clinicopathological profile of all cases of liver MINEN reported till date

**Year**	**Reference**	**Age (year) /sex**	**Symptoms**	**Co-existent** **hepatitis/** **etiology of** **hepatitis**	**Tumor size**	**Subtype**	**Tumor markers**	**Tumor components**	**Treatment received**	**Metastasis**	**Outcome survival**	**Region**
1984	Barsky et al. (10)	43/M	Right upper quadrant pain	HBV	NR	Combined	AFP ¥	HCC +Grade 1NET (carcinoid)	Palliative chemotherapy (adriamycin + 5-F/U)	Omental metastasis at presentation	Dead at 26 months	USA
1994	Artopoulos and Destuni (11)	69/M	Mild abdominal pain	HBV	10cms	Combined	Not mentioned	HCC + NEC	Surgical resection	No	NR	Europe
2000	Vora et al. (12)	63/M	Abdominal pain and jaundice	NK	10cms	Combined	NK	HCC +NEC	Surgical resection	No	Death at 1 month	Asia
2003	Ishida et al. (13)	72/M	None	HCV	3cms	Collusion	AFP ¥	HCC+NEC	Surgical resection	Regional lymph node metastasis	NR	Asia
2004	Yamaguchi et al. (14)	71/M	None	HCV	4.1cms	Combined	AFP ¥	HCC+NEC	Surgical resection	Metastasis (NEC) at pelvic bone in 5 months	NR	Asia
2006	Garcia et al. (15)	50/M	None	HCV	5.3cms	Collusion	AFP ¥	HCC+NEC	Surgical resection, TACE and palliative chemotherapy (doxorubicin + thalidomide + bevacizumab)	Liver recurrence in 4 months followed by regional LNs and peritoneal metastasis	Alive at 15 months	USA
2009	Yang et al. (16)	65/M	Intermittent epigastric pain	HBV	7.5cms	Combined	AFP not elevated	HCC+NEC	Surgical resection	Regional LN (NEC) at diagnosis. Local recurrence and adrenal metastasis in 3 months	Death at 12 months	Asia
2011	Tazi et al. (17)	68/M	None	HBV	4cms	Collusion	AFP ¥	HCC+NEC	Surgical resection, adjuvant chemotherapy (cisplatin + etoposide) x 4 cycles	None	Alive at 28 months F/U	Africa
2012	Hammedi et al. (18)	56/M	None	Neg HBV/HCV	Multifocal	Combined	AFP ¥	HCC+NEC	None	Regional nodal mets at presentation	Dead within 1 month	Africa
2012	Nakanishi et al. (19)	76/M	None	HCV/Alcohol	3cms	Combined	AFP ¥	HCC+NEC+ sarcomatous component	TACE followed by surgical resection	Skeletal metastasis (NEC) in 6 months	Dead at 7 months	Asia
2014	Aboelenen et al. (20)	51/M	Dull aching abdominal pain	HCV	20cms	Combined	AFP not elevated	HCC+NEC*	Liver transplant	None	Alive at 6 months F/U	Africa
2016	Nishino et al. (21)	72/M	None	HCV	2.5cms	Combined	NK	HCC+NEC	Surgical resection, palliative chemotherapy (cisplatin + etoposide)	Regional and non-regional LN metastasis in 1 week	Dead at 3 months	Asia
2016	Yun et al. (8)	68/F	None	HBV	2.5cms	Combined	AFP ¥	HCC+NEC	Surgical resection, palliative chemotherapy (cisplatin + etoposide) x 5 cycles	Distant recurrence in 6 months	Dead at 13 months	Asia
2017	Nomura et al. (2)	71/M	Not mentioned	HCV	4.1	Combined	AFP ¥	HCC+NEC	Surgical resection	Liver recurrence	Dead at 8.6 months	Asia
2017	Nomura et al. (2)	71/M	Not mentioned	HCV	3	Collusion	AFP ¥	HCC + NEC+ sarcomatous component	RFA followed by surgical resection	Liver recurrence	Dead at 2.6 months	Asia
2017	Nomura et al. (2)	58/M	Not mentioned	HBV	4.3	Combined	AFP ¥	HCC+NEC	Surgical resection	NR	Alive at 19.6 months follow up	Asia
2017	Nomura et al. (2)	50/M	Not mentioned	HBV	1.8	Combined	AFP ¥	HCC+NEC	Surgical resection	NR	Alive at 19.5 months follow up	Asia
2017	Nomura et al. (2)	63/M	Not mentioned	HCV	3	Combined	AFP ¥	HCC+NEC	Surgical resection	NR	Alive at 19.5 months follow up	Asia
2016	Baker et al. (22)	76/M	None	Not mentioned	5.5	Collusion	AFP ¥	HCC+NEC	Surgical resection, adjuvant chemotherapy (platinum-based regimen)	NR	Alive, F/U duration NR	USA
2017	Beard et al. (23)	19/M	Hepatomegaly	Not mentioned	25cms	Combined	AFP not elevated, Ca 19.9 ¥	HCC+ cholangio carcinoma + NEC	Surgical resection followed by adjuvant chemotherapy (gemcitabine + cisplatin) x 6 cycles, Palliative chemotherapy with (capecitabine + temozolomide)	Regional and non-regional LN recurrence in 4 months (all 3 components with NEC*)	Alive at 8 months	USA
2017	Liu et al. (24)	65/M	Right upper quadrant pain	HCV	4.3cms	Collusion	AFP¥	HCC+NEC	Surgical resection	Regional lymph node metastasis (NEC) at presentation	Dead at 1.3 months	Asia
2017	Okumura et al. (25)	70/M	None	HCV	11cms	Combined + collusion	AFP not elevated	HCC+NEC	TACE and PTPE followed by surgical resection, palliative radiation, palliative sorafenib	Lymph node and skeletal metastasis in 1 month	Death at 3 months	Asia
2017	Lu et al. (26)	65/M	Right upper quadrant pain	NR/NR	14	Combined	AFP ¥	HCC+NEC	Hospice	Rapid progression and distant metastasis within 1 month	NR	USA
2018	Kwon et al. (27)	44/M	None	HBV	10.5	Combined	AFP NR/ectopic thyroid hormone	HCC+ cholangio carcinoma+ NEC	Surgical resection, adjuvant 5-F/U and radiation	Distant metastasis in 2 months	Dead at 2 months	Asia
2018	Yilmaz et al. (28)	56/M	Abdominal distention related to ascites	Alcoholic , negative for HBV and HCV	1.7	Collusion	AFP not elevated	HCC+NEC	Liver transplant	NR	Alive at 10 months follow-up	Turkey
2020	Ikeda et al. (29)	79/M	Increased hepatobiliary enzyme levels	negative for HBV and HCV		Combined	AFP ¥	HCC+NEC	Surgical resection	Recurrence at 4 months	Dead at 4 months	Asia
2020	Jahan et al. (4)	50/M	None	Alcohol/HCV	2.7	Combined	AFP not elevated	HCC+NEC	Surgical resection, 90Y radioembolization, palliative radiation, palliative chemotherapy (cisplatin+ etoposide) x 8 cycles, and nivolumab x 2 cycles	Local recurrence in 13 months and distant recurrence in 17 months	Dead at 33 months	USA
2020	Kaneko et al. (9)	70/F	Abdominal pain	NR	Multiple tumors	Combined	NR	(MINEN) Cholangio carcinoma+ NEC	Treated with Gemcitabine,S1 and octreotide acetate,followed by transcatheter arterial embolisation	Subsequently developed GB mass which showed carcinosarcoma	Alive at 44 months	Asia
2021	Lan et al. (5)	39/M	Anorexia	HBV	20cms	Combined	AFP ¥	HCC+NEC	TACE+Surgical excision followed by cisplatin and etoposide basedn chemotherapy	No recurrence at 6 month follow up	NR	Europe
2022	Tanaka et al. (6)	70/M	None	HBV	5cms	Combined	AFP/PIVKA II¥¥	HCC+NEC	Surgical resection	No recurrence at 12 months	NR	Asia
2023	Shin et al. (7)	63/M	None	HBV/HCV negative	7.3cms	Combined	AFP¥¥¥/PIVKA II¥	Poorly diff HCC+NEC*	Surgical resection followed by chemotherapy with cisplatin and etoposide followed by radiotherapy and addition of irinotecan once adrenal mets developed 6 months after surgical resection.	Recurrence at 6 months in adrenal followed by other lesions in liver lung and brain	Died at 12 months	Asia
2023	Present case	73/M	Diarrhea	HBV/HCV negative	5cms	Combined	AFP normal/PIVKA II ¥¥	HCC (Grade III+NEC)	Surgical resection with ERBT (4500cGy over 1week)	FDG avid lung nodule with bilateral adrenal metastasis.	Alive after 15 months	Asia
2023	Present case	44/F	Pedal edema and ascites	HBV/HCV negative	Multifocal	Combined	AFP normal/PIVKA ¥	HCC+NEC	Liver transplantation with 4 cycles of cisplatin and etoposide regimen.	D6-D8 extradural lesion likely metastasis.	Alive at 12 months	Asia

**M:** male, **F:** female, **FU:** fluorouracil, **HBV:** hepatitis B virus, **HCC: **hepatocellular carcinoma, **HCV:** hepatitis C virus, **LN:** lymph node, **NEC:** neuroendocrine carcinoma, **NK:** not known to author, **NR: **not reported, **PTPE:** percutaneous transhepatic portal vein embolization, **TACE:** trans-arterial chemoembolization, **90** **Y:** yttrium-90 microsphere, **(**¥**):** elevated.

Most of the liver tumors including NET/MINEN present as solitary, well-circumscribed solitary masses (1) as was seen in this review. The size of the solitary tumors ranged from 1.8 cm to 25 cm with a mean size of 9.94 cm, excluding multifocal tumors. The exact pathogenesis of mixed HCC-NEC is unknown. There are two predominant hypotheses in regard to the origin of this rare type of tumor: 1) under certain circumstances well or moderately well-differentiated HCC changes phenotype to NEC (4,13), and 2) hepatic stem cells differentiate to both HCC and NEC components (23).

While going through the literature, all the cases including ours were reported as HCC pre-operatively because of arterial phase enhancement and delayed venous wash out. AFP and PIVKA-II are the commonly used biomarkers for HCC. Alpha-fetoprotein is found to be elevated in 70 - 90% of cases with a sensitivity of 60% and specificity of 90% (30). However when PIVKA-II and AFP are combined, the diagnostic power improves significantly compared to either AFP or PIVKA-II (p<0.05) (31). Out of 33 cases of MINEN, AFP levels were raised in 21 cases (72.4%), normal in 8 cases (27.5%), and were not available in 4 cases. PIVKA-II on the other hand was not done in a significant number of cases and was available only in 4 cases. PIVKA was increased in all the four cases including our cases.

Most of the cases underwent surgical resection. In 30 (90.8%) cases, surgical excision was done, out of which 3 patients including our case underwent liver transplantation. In three cases (10,18,26), only palliative treatment was given. On histology, 29 cases (87.85%) presented with a mixture of HCC and neuroendocrine carcinoma, 2 cases (6.4%) presented with a mixture of HCC, neuroendocrine carcinoma, and a sarcoma component, and 2 cases (6.4%) presented with a mixture of HCC, cholangiocarcinoma, and NEC.

In the literature, mixed HCC-NECs have been broadly divided into two categories based on their histological arrangement. **1)** **Combined type:** where HCC and NEC components are in contact with each other, and they often have a transitional zone where both cell types are admixed with each other. Nomura et al. described them as ‘transitional type’ (2). **2) Collusion type: **where HCC and NEC components create distinctive tumors without any transitional zone. HCC and NEC component are usually separated by fibrous septa. Nomura et al. described them as ‘separate type’ (2). Sometimes collusion types of tumor components could be found in different segments of the liver (4). Most of the cases sited in the literature are the combined type (77.4%), including our cases.

Besides classical hepatocellular carcinoma (HCC) and cholangiocarcinoma (CC), combined and intermediate forms of liver cancer exist and can express stem-cell markers like nuclear cell adhesion molecule (NCAM-1/CD56), c-kit (CD117), or epithelial cell adhesion molecule (EpCAM) together with high proliferative activity. Liver tumors with progenitor-cell features are associated with an unfavorable prognosis (7,32). In our two cases, although positivity for CD56 and high Ki 67 index was seen no positivity for CD117 was noticed. Besides this, our cases showed positivity for synaptophysin.

MINEN of the liver has a very poor prognosis as local or distant recurrence is common after surgical resection and usually is fatal. The role of adjuvant chemotherapy following surgical resection is not clear. Going through the literature, various treatment modalities have been used, with systemic chemotherapy with cisplatin and etoposide used in most cases. Liver transplantation was used in 2 cases and both cases (20,28) were doing fine when reported, without recurrence. Our patient with liver transplantation is on follow up and alive after 1 year. Mean survival ranged from 1 month to 33 months. No significant differences were seen in clinicopathological profile of these cases, which could tally for this wide survival range. Interestingly one case of mixed cholangiocarcinoma and NEC (9) behaved very well and was alive at 44 months.

To conclude, MINEN is a rare tumor of the liver and has a poor prognosis. Though each component should be 30% as per the latest guidelines (3), there have been cases (4) in which the NEC component was even less than 1% and was retrospectively diagnosed when the patient presented with metastasis of the neuroendocrine component, emphasizing the need for reporting of the NEC component irrespective of the percentage, as it renders a poor prognosis and brings the role of combined chemotherapy into play.

## Conflict of Interest

The authors have no conflicts of interest to declare.
